# The Acid Soluble Disulphide and Mixed Disulphide Levels of Some Normal Tissues and Transplanted Tumours

**DOI:** 10.1038/bjc.1970.72

**Published:** 1970-09

**Authors:** G. Calcutt, S. M. Ting

## Abstract

The acid soluble disulphides and mixed disulphides of a range of normal rat and mouse tissues and a number of transplanted rat or mouse tumours were measured. The result were considered in relation to other workers' data. It is noted that more radioresponsive tissues have higher levels than the more radioresistant tissues.


### THE ACID SOLUBLE DISULPHIDE AND MIXED DISULPHIDE LEVELS OF SOME NORMAL TISSUES AND TRANSPLANTED TUMOURS

G. CALCUTT AND S. M. TING

*From the Department of Cancer Research, Mount Vernon Hospital and the Radium Institute, Northwood, Middlesex*

Received for publication May 13, 1970

**SUMMARY.—The acid soluble disulphides and mixed disulphides of a range of normal rat and mouse tissues and a number of transplanted rat or mouse tumours were measured. The results were considered in relation to other workers' data. It is noted that the more radioresponsive tissues have higher levels than the more radioresistant tissues.**

For a long time it was generally considered that the level of acid soluble disulphides in tumours was very low. Tarnowski, Barclay, Mountain, Nakamura, Satterwhite and Solney (1965) used a newly described reduction system—by means of sodium borohydride—and concluded that the concentration of acid soluble disulphides was comparable with the concentration of acid soluble thiols. This conclusion, however, was not warranted since Tarnowski *et al.* carried out their reductions on whole homogenates of tumours and then after deproteinisation estimated the thiol level for a comparison with that derived from a similar system without the reducing step. They therefore measured acid soluble disulphides plus any small acid soluble thiol groups broken off from proteins during the reduction process; that is the usually described " mixed disulphides ".

Based on data derived from experiments with the Ehrlich ascites tumour Révész (1969) has concluded that the acid soluble disulphides exist at a low level as compared with the mixed disulphides.

To clarify this situation we have undertaken a new series of measurements of both acid soluble and mixed disulphides in a number of transplanted mouse and rat tumours. For comparison similar measurements have been made on a variety of normal mouse and rat tissues.

### METHODS

### *Acid soluble disulphides*

Having considerable experience of the measurement of sulphydryl (–SH) groups by the technique described by Calcutt and Doxey (1959) and Calcutt, Doxey and Coates (1960) the system of measuring the difference between acid soluble thiols with and without reduction was preferred. Test runs involving a variety of reducing systems gave unsatisfactory results. The only method found to be repeatable and reliable was to homogenise a weighed amount of tissue in the presence of EDTA and $\alpha\alpha$ dipyridyl as described by Calcutt and Doxey (1962) and then to precipitate proteins with trichloracetic acid. After removal of the protein the acid solution was divided, one portion being used for a straightforward thiol estimation whilst the other was adjusted to pH 6·8 and reduced with sodium sulphite before thiol estimation. When known amounts of highly purified oxidised glutathione (GSSG) were added to this system recoveries consistently fell within the range of 97–104%.

### *Mixed disulphides*

The usual method of reduction of precipitated washed protein with excess sodium borohydride was used. After termination of the reduction by the addition of excess acid the protein was filtered off and sulphydryl estimated on the filtrate. Reduction was carried out for fifteen minutes at room temperature. Prolongation of the time or increase in temperature as used by Révész and Modig (1965) resulted in losses of the released –SH groups, this, probably, arising from the instability of thiols in alkaline solutions.

### RESULTS

Work by Beck, Rieck and Duncan (1958) and Calcutt (1964, 1967) has shown that well-defined diurnal variations occur in intracellular thiol levels, whilst Calcutt and Ting (1969) have found similar diurnal fluctuations in levels of disulphides in rat liver, spleen and kidney. The present results are therefore based on measurements made at a standard time of day, usually between 10 and 11 a.m. so as to avoid any diurnal variations.

Detailed figures are given in Tables I–IV. In all cases the disulphide values were calculated as their equivalent as free –SH.

TABLE I.—*Acid Soluble Disulphide and Mixed Disulphide Levels of Normal Tissues from 15-week-old Sprague-Dawley Rats*

(In this and subsequent tables all measurements are expressed as μg. –SH per 100 mg. wet weight of tissue.)

| | Acid soluble disulphides | | | Mixed disulphides | | |
|---|---|---|---|---|---|---|
| Tissue | Number of samples | Mean SS | Range | Number of samples | Mean SS | Range |
| Male rats | | | | | | |
| Liver | 14 | 1·78 | 0–6·4 | 20 | 0·57 | 0–2·1 |
| Spleen | 14 | 5·92 | 0–14·1 | 20 | 1·53 | 0–4·7 |
| Kidney | 13 | 0·94 | 0–2·6 | 20 | 0·57 | 0–1·1 |
| Bone marrow | 20 | 11·1 | 0–19·2 | 12 | 1·79 | 0–3·6 |
| Thymus | 20 | 2·74 | 0–7·8 | 12 | 0·75 | 0–1·2 |
| Peyer's patches | 19 | 5·26 | 0–14·7 | 12 | 1·01 | 0–2·0 |
| Intestine | 13 | 2·6 | 0–8·9 | 11 | 0·67 | 0–2·6 |
| Female rats | | | | | | |
| Liver | 17 | 2·0 | 0–8·1 | 12 | 0·44 | 0–1·0 |
| Spleen | 17 | 10·8 | 0–21·4 | 12 | 0·89 | 0–1·5 |
| Kidney | 18 | 0 | — | 12 | 0·52 | 0–1·2 |
| Bone marrow | 14 | 7·8 | 0–15·3 | 12 | 2·0 | 0–5·4 |
| Thymus | 11 | 3·98 | 0–11·9 | 12 | 0·85 | 0–3·7 |
| Peyer's patches | 15 | 6·44 | 0–18·2 | 12 | 1·35 | 0–3·2 |
| Intestine | 4 | 0·75 | 0–3·1 | 12 | 1·11 | 0–3·9 |

TABLE II.—*Acid Soluble Disulphide and Mixed Disulphide Levels of Normal Tissues from 12-week-old BALB/c Mice*

| Tissue | Acid soluble disulphides: Number of samples | Mean -SS- | Range | Mixed disulphides: Number of samples | Mean -SS- | Range |
|---|---|---|---|---|---|---|
| Male mice | | | | | | |
| Liver | 15 | 0 | — | 9 | 1·9 | 0·7–3·9 |
| Kidney | 15 | 0 | — | 4 | 0·3 | 0–0·1 |
| Spleen | 6 | 2·45 | 0–7·8 | 8 | 3·1 | 2·6–4·3 |
| Female mice | | | | | | |
| Liver | 15 | 13·3 | 5·2–23·0 | 15 | 2·5 | 2·1–3·1 |
| Kidney | 5 | 0 | — | 8 | 3·3 | 0·9–4·5 |
| Spleen | 8 | 16·7 | 0–25·0 | 8 | 3·5 | 2·6–4·3 |

TABLE III.—*Acid Soluble and Mixed Disulphides in Transplanted Rat Tumours*

| Tumour | Host strain | Host sex | Acid soluble disulphides: Number of samples | Mean -SS- | Range | Mixed disulphides: Number of samples | Mean -SS- | Range |
|---|---|---|---|---|---|---|---|---|
| M.V.Y. | Wistar | M | 10 | 4·24 | 0–11·9 | 11 | 1·16 | 0–3·3 |
| (sarcoma) | Wistar | F | 7 | 9·36 | 0–13·5 | — | — | — |
| Walker | Wistar | M | 26 | 2·2 | 0–7·6 | 23 | 0·44 | 0–0·9 |
| (carcinosarcoma) | Wistar | F | 7 | 3·18 | 0–15·3 | 7 | 0·45 | 0–0·6 |
| S.14 (adenocarcinoma) | Sprague-Dawley | M | 4 | 2·68 | 0–9·6 | 4 | 1·33 | 0·8–1·8 |

TABLE IV.—*Acid Soluble Disulphides and Mixed Disulphides in Mouse Tumours*

| Tumour | Host strain | Host sex | Acid soluble disulphides: Number of samples | Mean -SS- | Range | Mixed disulphides: Number of samples | Mean -SS- | Range |
|---|---|---|---|---|---|---|---|---|
| S.180 | BALB/c | M | 12 | 1·3 | 0–6·4 | 7 | 1·05 | 0·1–2·0 |
| (sarcoma) | BALB/c | F | 10 | 0 | — | 18 | 1·52 | 0·4–2·6 |
| PL.64 (carcinoma) | BALB/c | F | 8 | 1·4 | 0–8·5 | 5 | 1·24 | 0·4–1·9 |
| ADJ/PC5 | BALB/c | M | 11 | 9·4 | 0–29·0 | 13 | 2·28 | 0–5·3 |
| (plasma cell) | BALB/c | F | 6 | 0 | — | 10 | 1·22 | 0–2·4 |
| Bp 64/XII | BALB/c | M | 3 | 0·56 | 0–1·4 | 4 | 0·15 | 0–0·6 |
| (sarcoma) | BALB/c | F | 3 | 6·0 | 1·5–9·8 | 5 | 1·73 | 1·0–2·8 |
| Bp 65/2 (sarcoma) | CBA | M | 9 | 0 | — | 3 | 1·15 | 0·4–1·9 |

### DISCUSSION

In common with many other biochemical measurements these results have shown a wide range of variation, this even applying to apparently similar portions of tissues from different animals. This is different from the findings of Tarnowski *et al.* (1965) who only found limited ranges of variation. In some cases it has not been found possible to detect any disulphide or mixed disulphide in a particular tissue. Since acid soluble thiols have always been found in all tissues this would suggest a lack of regular relationship between thiol and disulphide levels. This conclusion had already been reached on other grounds by Calcutt and Ting (1969).

When acid soluble disulphide and mixed disulphide levels for any one tissue are compared no coherent picture emerges. Sometimes one is higher, sometimes the other. Under these circumstances the case of Ehrlich ascites cells described by Révész (1969) must be considered as a particular case and not representative of any overall pattern.

Comparison of the figures for corresponding tissues from male and female animals shows no consistent pattern. This applies to both acid soluble disulphides and to mixed disulphides. In the case of mouse tumours transplants into male hosts show higher figures than those into females. Rat tumours show a reversal of this picture. This would seem to highlight the necessity for careful consideration of the host sex when associating tumour biochemistry with mechanisms of action of drugs or irradiation.

When the levels of either acid soluble disulphides or mixed disulphides of various tissues are compared it is evident that certain tissues, *e.g.*, bone marrow or spleen, have very much higher levels than others. In fact it is the more radio-responsive tissues which show the higher levels. Further examination of this point is needed since the question of the role of disulphides in radiation response does not appear to have been raised previously.

Although this study has not confirmed some recent work it is in agreement with the conclusion of Tarnowski *et al.* (1965) that very low levels of disulphides previously reported are not a true picture. Further it has raised some new issues which justify further investigation.

The expenses of this work were defrayed from a block grant from the British Empire Cancer Campaign for Research.

### REFERENCES

Beck, L. V., Rieck, Virginia D. and Duncan, Blendina—(1958) *Proc. Soc. exp. Biol. Med.*, **97**, 229.

Calcutt, G.—(1964) *Br. J. Cancer*, **18**, 197.—(1967) *Naturwissenschaften*, **54**, 120.

Calcutt, G. and Doxey, D.—(1959) *Expl Cell Res.*, **17**, 542.—(1962) *Br. J. Cancer*, **16**, 562.

Calcutt, G. Doxey, D. and Coates, Joan—(1960) *Br. J. Cancer*, **14**, 746.

Calcutt, G. and Ting, S. M.—(1969) *Naturwissenschaften*, **56**, 418.

Révész, L.—(1969) In 'Radiation Damage and Sulphydryl Compounds', p. 125. International Atomic Energy Agency, Vienna.

Révész, L. and Modig, H.—(1965) *Nature, Lond.*, **207**, 430.

Tarnowski, G. S., Barclay, R. K., Mountain, Isabel M., Nakamura, M., Satterwhite, H. G. and Solney, E. M.—(1965) *Archs Biochem. Biophys.*, **110**, 210.


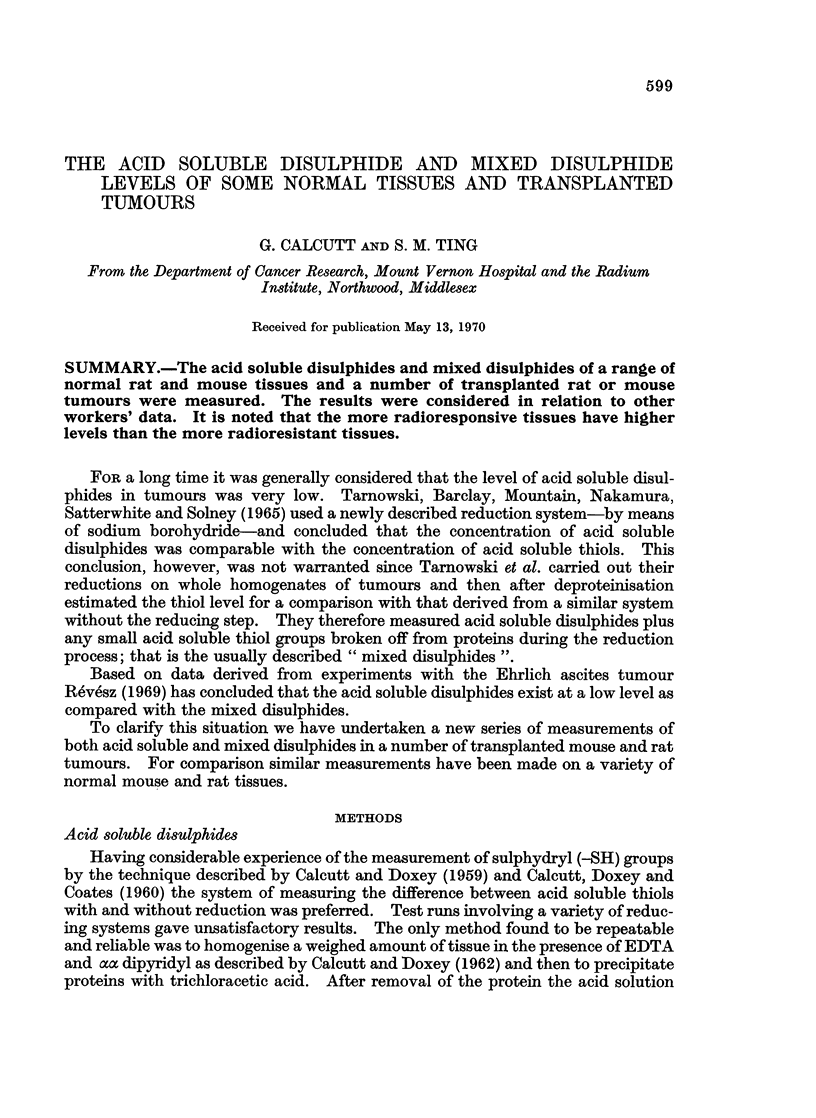

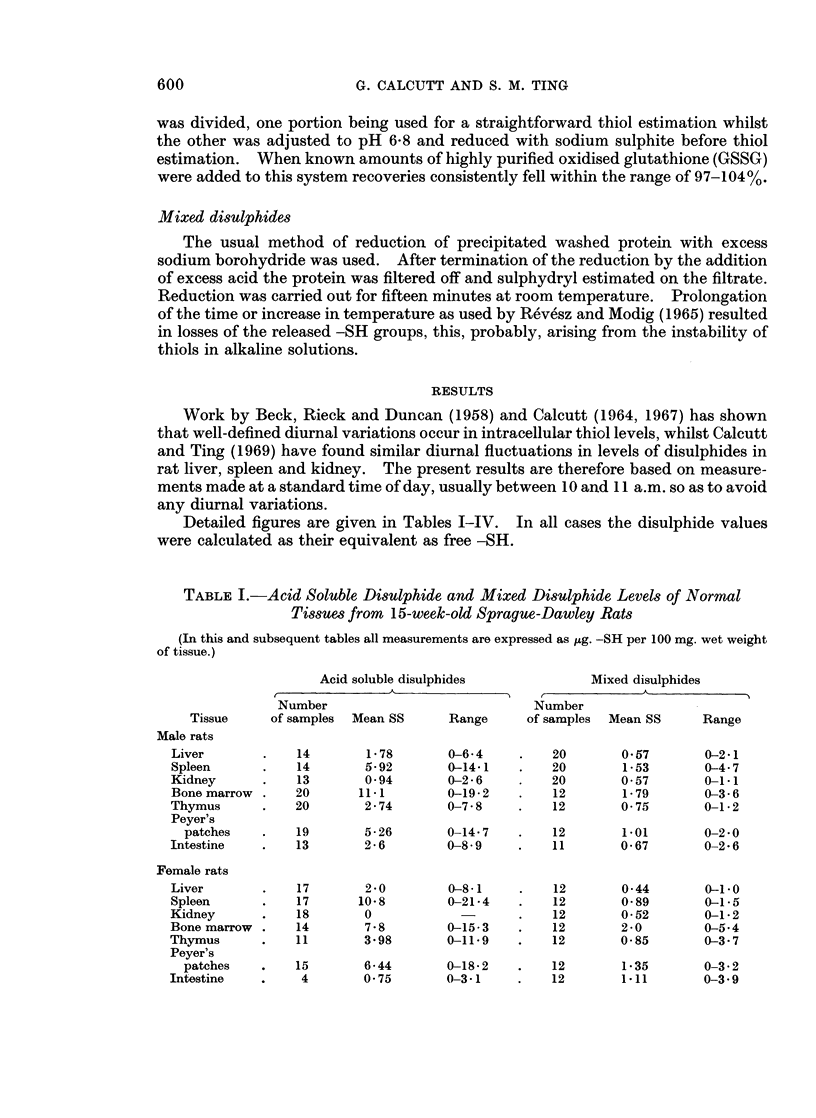

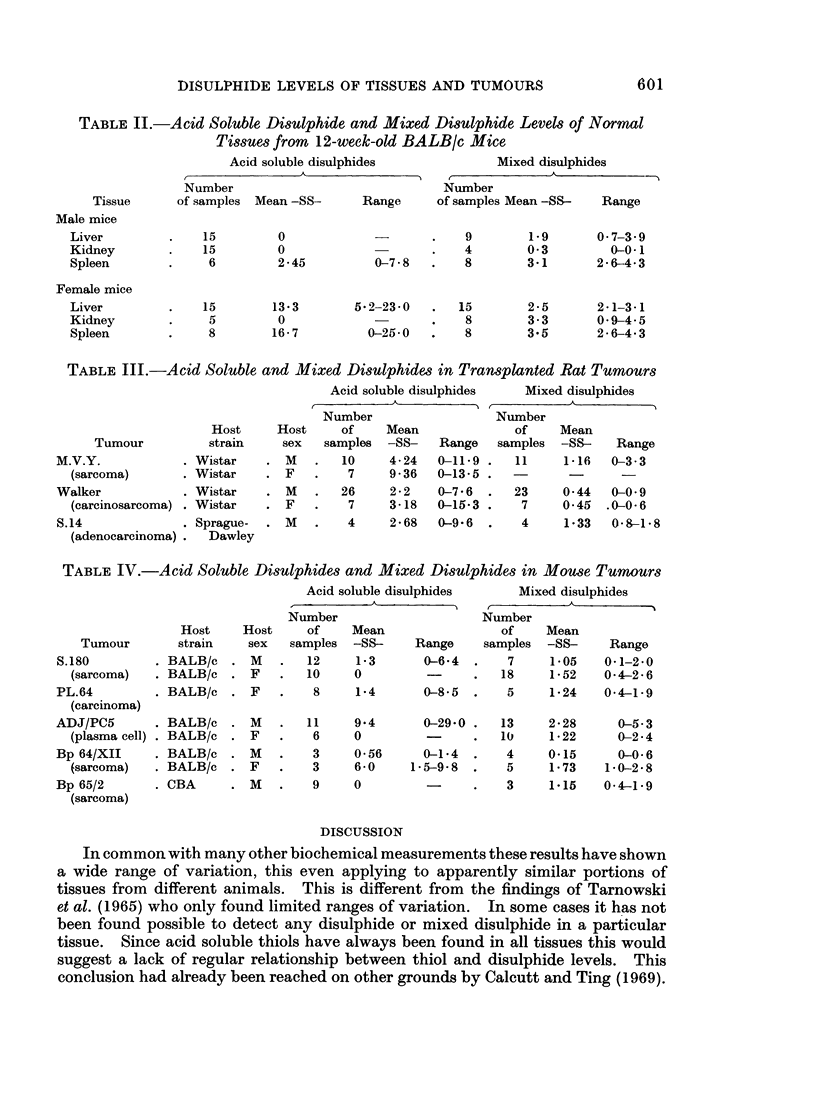

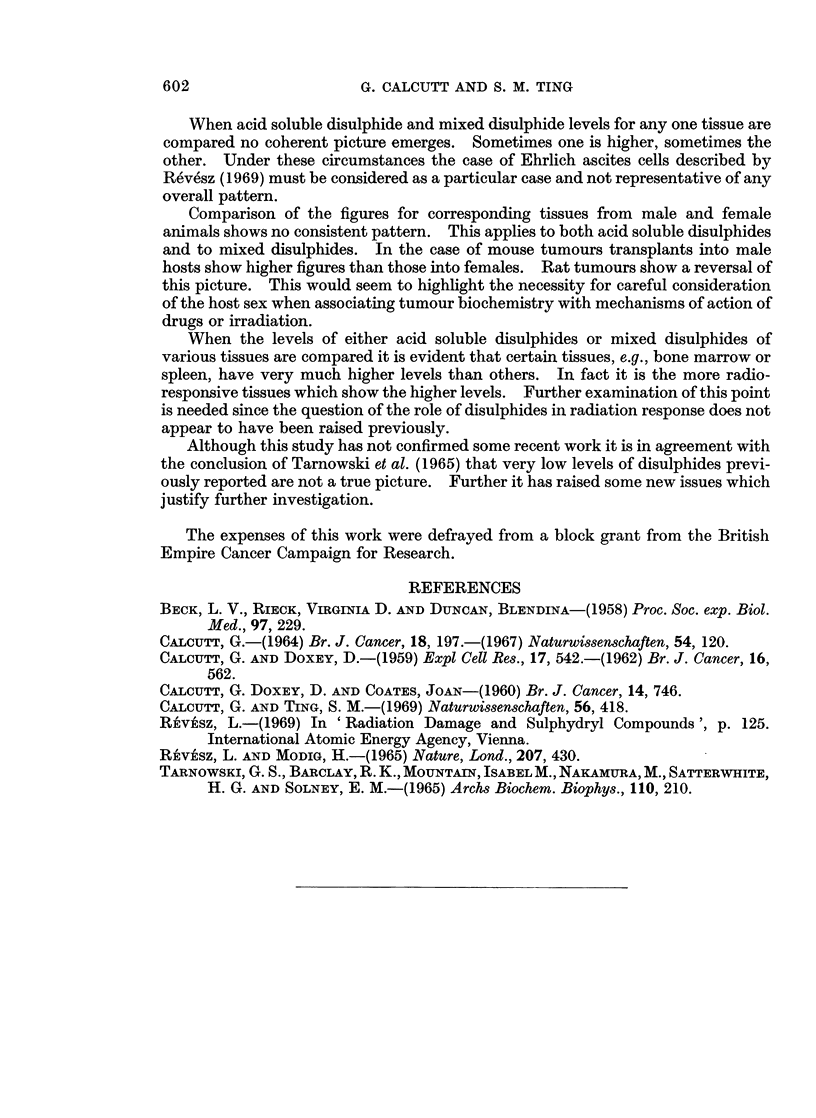


## References

[OCR_00329] BECK L. V., RIECK V. D., DUNCAN B. (1958). Diurnal variation in mouse and rat liver sulfhydryl.. Proc Soc Exp Biol Med.

[OCR_00337] CALCUTT G., DOXEY D., COATES J. (1960). The effects of some chemical carcinogens upon the-SH levels of target and non-target tissues.. Br J Cancer.

[OCR_00335] CALCUTT G., DOXEY D. (1959). The measurement of tissue-SH.. Exp Cell Res.

[OCR_00344] Révész L., Modig H. (1965). Cysteamine-induced increase of cellular glutathione-level: a new hypothesis of the radioprotective mechanism.. Nature.

[OCR_00346] TARNOWSKI G. S., BARCLAY R. K., MOUNTAIN I. M., NAKAMURA M., SATTERWHITE H. G., SOLNEY E. M. (1965). DETERMINATION OF ACID-SOLUBLE THIOLS AND DISULFIDES IN TRANSPLANTED ANIMAL TUMORS.. Arch Biochem Biophys.

